# MicroRNA in lung cancer—a novel potential way for early diagnosis and therapy

**DOI:** 10.1007/s13353-023-00750-2

**Published:** 2023-02-23

**Authors:** Magdalena Frydrychowicz, Łukasz Kuszel, Grzegorz Dworacki, Joanna Budna-Tukan

**Affiliations:** 1 Department of Clinical Immunology, Poznan University of Medical Sciences, 60-806 Poznan, Poland; 2 Department of Medical Genetics, Poznan University of Medical Sciences, 60-806 Poznan, Poland; 3 Department of Histology and Embryology, Poznan University of Medical Sciences, 61-781 Poznan, Poland

**Keywords:** miRNA, Liquid biopsy, Cancer biomarkers, Lung cancer, Prognostic value, Immunotherapy

## Abstract

Lung cancer is the most common cause of cancer-related deaths in the world. One of the reasons of poor prognosis and high mortality of lung cancer patients is the diagnosis of the disease in its advanced stage. Despite innovative diagnostic methods and multiple completed and ongoing clinical trials aiming at therapy improvement, no significant increase in patients’ long-term survival has been noted over last decades. Patients would certainly benefit from early detection of lung cancer. Therefore, it is crucial to find new biomarkers that can help predict outcomes and tumor responses in order to maximize therapy effectiveness and avoid over- or under-treating patients with lung cancer. Nowadays, scientists’ attention is mainly dedicated to so-called liquid biopsy, which is fully non-invasive and easily available method based on simple blood draw. Among common liquid biopsy elements, circulating tumor nucleic acids are worth mentioning. Epigenetic biomarkers, particularly miRNA expression, have several distinct features that make them promising prognostic markers. In this review, we described miRNA’s involvement in tumorigenesis and present it as a predictor of cancer development and progression, potential indicator of treatment efficacy, and most importantly promising therapeutic target.

## Introduction

Lung cancer is the main cause of cancer death worldwide. The GLOBOCAN 2018 database estimated 1.76 million deaths and 2.09 million (11.6%) new cases associated with this malignancy in 2018 (Wu et al. 2019a; Ferlay et al. 2019).

According to histological characteristics, lung cancer is divided into two main types: small cell lung cancer (SCLC) and non-small cell lung cancer (NSCLC). Fifteen percent of lung cancer cases are SCLC, mainly resulting from tobacco smoking. In turn, NSCLC—which makes up 85% of all lung cancers—is further categorized into adenocarcinoma (LUAD), squamous cell carcinoma (LUSC), and large cell carcinoma, based on origin the tumor origin and the type of cellular pathology observed (Lemjabbar-Alaoui et al. 2015).

Lung cancer development is a complicated process, influenced by both genetic and environmental factors (Wu et al. 2019a).

The majority (60–80%) of lung cancer patients are diagnosed at an advanced stage, despite extensive clinical trials, cutting-edge diagnostic methods, and improved supportive treatment. Therefore, over the past few decades, long-term survival has not seen a significant increase (Lemjabbar-Alaoui et al. 2015).

Early identification of lung cancer is vital for a considerable reduction in cancer morbidity and mortality. To enhance lung cancer diagnosis, we must first gain a full understanding of carcinogenesis, not only genetically, but also epigenetically. Hence, it is crucial to find new biomarkers that can help predict outcomes and tumor responses in order to maximize therapy effectiveness and avoid over- or under-treating patients with lung cancer. miRNA expression is one of the distinctive epigenetic biomarkers that have potential for use as a potential effective prognostic marker (Larrea et al. 2016).

miRNAs are a family of small noncoding RNAs (21–25 that can inhibit messenger RNA (mRNA) translation and promote mRNA degradation by base pairing to complementary sites of target mRNAs. Through posttranscriptional control and transcript degradation, miRNAs aid development, body patterning, stem cell differentiation, and tissue identity (Weiss and Ito 2017).

The human genome currently has 2654 mature miRNAs sequences, according to the miRBase (miRNA sequence database) (version 22.1, October 2018) (http://www.mirbase.org/) (Loh et al. 2019; Kozomara et al. 2018).

There are several steps involved in the synthesis of miRNAs from miRNA genomic sites. Pre-miRNAs, the ancestors of miRNAs, are typically described as double-stranded stem-loop structures with polyadenylated caps on their ends. These transcripts are processed by an RNase III family member, the Drosha enzyme, in the nucleus to create pre-miRNAs, which are small RNA molecules with a hairpin shape, a 5′ phosphate group, and a 3′ double nucleotide (Bhaskaran and Mohan 2014; Lee et al. 2003).

**Fig. 1:**
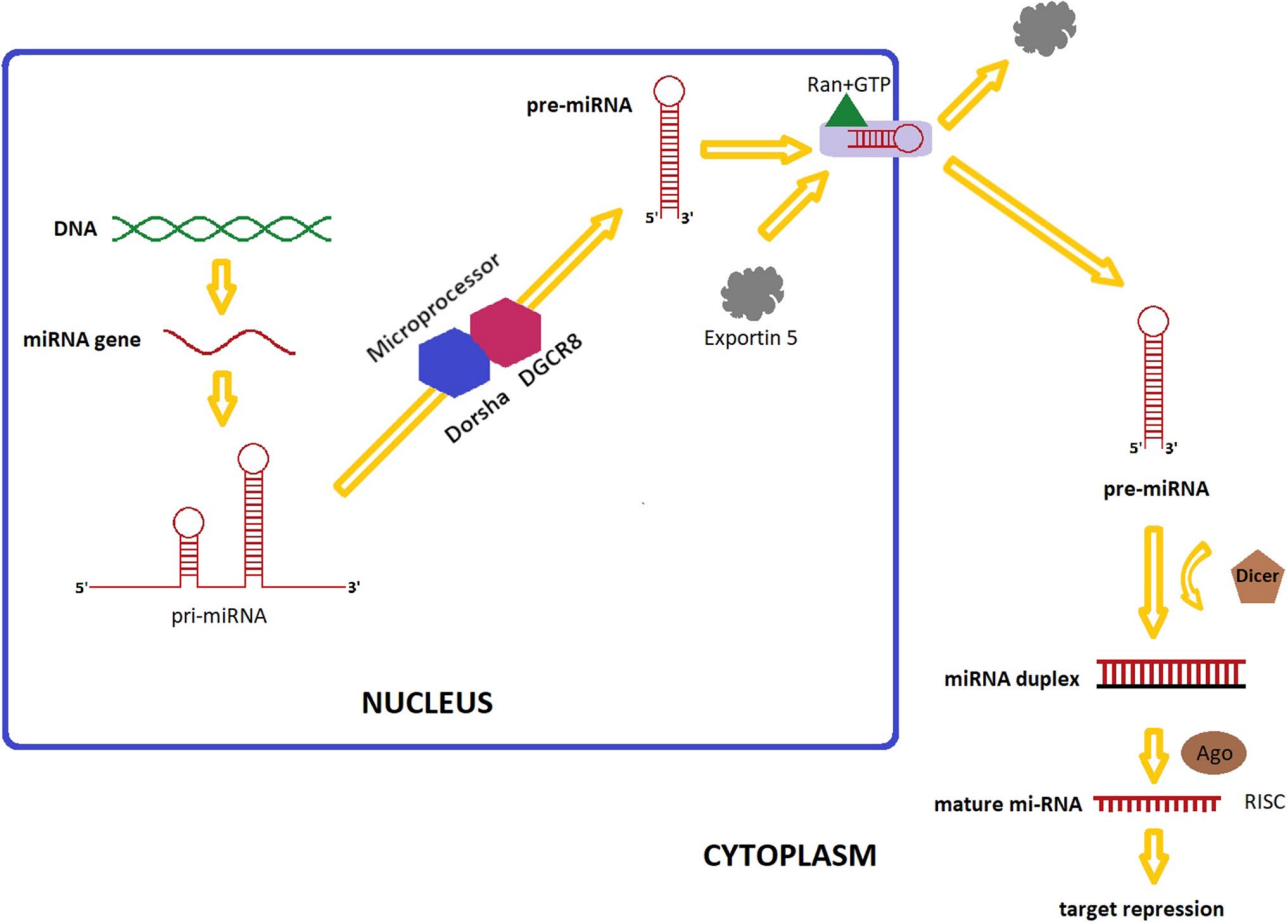
A schematic miRNAs biogenesis

Pre-miRNAs are exported to the cytoplasm by exportin-5, and enzymes like the Dicer ribonuclease complex use them to create microRNAs of 21–23 nucleotide sequences (Yi et al. 2003; Macrae et al. 2006). This is a component of the RISC (RNA-induced silencing complex) that unravels miRNA chains, cleaves them, and releases the guide and “passenger” chains. The complex’s Argonaute 2 endonuclease interacts with the complementary regions of mRNA to cause degradation and/or translational inhibition of the latter (Lee et al. 2003; Liu et al. 2004; Hutvagner and Simard 2008; Ambros 2004; Whitehead et al. 2009). A summary of the miRNA biogenesis is shown in Fig. 1.

miRNA biogenesis begins in the nucleus. miRNA genes are transcribed by RNA-polymerase II-dependent (RNAPII). The primary transcript is created (pri-miRNAs). The initial processing of the primary transcript is mediated by microprocessor complex (Drosha-DGCR8 complex) that generates pre-miRNAs. The pre-miRNAs is exported by Exportin 5/RanGTP complex out of the nucleus into the cytosol, where further processing by a second RNase III enzyme, Dicer takes place, and this leads to the production of a miRNA duplex. Finally, miRNA duplex is loaded into the Argonaute (AGO) family of proteins to form a miRNa-induced silencing complex (miRISC). One of the strands of the miRNA duplex is removed, resulting in a single-stranded miRNAs.

## miRNA testing

While plasma and serum are the most frequently used bodily fluids for a miRNAs analysis, urine and saliva can also be used. When it comes to methods of analysis, real-time reverse transcription PCR (RT-PCR, qRT-PCR) is the most popular in miRNA study. With the help of specialized microplates and specific primers, this technique’s high specificity and sensitivity enable the detection of a single miRNAs among hundreds of others. Furthermore, TaqMan Cards® (Life Technologies) are also a commonly used platform used for microRNA identification. To reverse transcribe miRNAs, stem-loop primers are used in this method. Conventional primers are used to amplify cDNA, while tagged fluorophores added to the reaction medium allow for real-time quantification. An alternative technique is based on polyadenylation of all miRNAs, addition of an antisense primer with a poly(T) motif at both the 5′ and 3′ ends, reverse transcription, and amplification of the results using a sense primer. The SYBR green fluorescent dye is most commonly used to identify the products (Lampignanoa et al. 2020; Kloten et al. 2019; Lu et al. 2018a; Pozniak et al. 2022; Mahjoob et al. 2022). Moreover, samples taken from the bodily fluids can be sequenced using either next-generation sequencing (NGS) or a microarray analysis (Pozniak et al. 2022).

NGS combines the techniques for DNA or RNA nucleotide sequence determination, making it feasible to concurrently identify many areas of the genome. NGS is carried out via several oligonucleotide ligations or cycles of polymerase-induced chain elongation. Nucleotide sequences of up to hundreds of megabases and gigabases can be produced in a single working cycle. NGS has the benefit of discovering novel miRNAs, but it is less efficient and less cost-effective than microarrays (Lampignanoa et al. 2020; Lu et al. 2018a; Pozniak et al. 2022).

Thousands of miRNAs can be examined at once using microarray analysis. Fluorescent probes are used to label the miRNA samples, followed by hybridization with hundreds or thousands of miRNAs that have been covalently immobilized on a solid platform (microchip), with subsequent detection of samples. LNA (locked nucleic acid) sequences found in the hybridization probes are utilized to standardize the melting temperatures of the miRNAs. This method makes it possible to identify miRNAs with comparable profiles and reduces variations in probe melting temperatures. However, this quantitative analytical technique can be technically challenging, making RT-PCR a common addition to it (Lu et al. 2018a; Pozniak et al. 2022; Montecalvo et al. 2012; Nakamura et al. 2016; Precazzini et al. 2021).

## MicroRNAs linked to lung cancer

Widespread evidence of an aberrant miRNA expression profile in cancer cells has been established. Almost all stages of carcinogenesis, including cell development, differentiation, proliferation, angiogenesis, apoptosis, invasion, and metastasis, are regulated by miRNAs. Mutations, methylation, deletion, or amplification of the miRNA-coding regions can all lead to changes in expression (Zhou et al. 2017; Egger et al. 2004; Negrini et al. 2009; Ganju et al. 2017).

Numerous miRNA genes are found in fragile sites or genomic areas associated with cancer. miRNAs are extremely vulnerable to genomic alterations in solid tumors and hematological malignancies (Zhou et al. 2017; Egger et al. 2004).

The analysis of miRNAs’ express profiles enables the distinction between healthy and cancerous tissues, identifies the origin of the tissue, and provides extremely accurate information on the subtype of a certain cancer. Additionally, miRNAs can be used to predict how a patient will respond to treatment and how the disease will progress (Palma et al. 2022; Lee and Dutta 2009; Shao et al. 2019).

Furthermore, different studies also demonstrated the importance of inflammation promotion, genomic instability, and mutations in cancer development. The combination between genetic predictions and epigenetic alternation is crucial for each phase in cancer biology, and miRNAs play a key role in each hallmark (Hanahan and Weinberg 2011; Langevin et al. 2015).

Lung cancer typically exhibits dysregulation of miRNAs, which can negatively influence the expression of hundreds of mRNA targets (Langevin et al. 2015).

miRNAs that frequently exhibit dysregulated expression in lung cancer have already been identified in two meta-analyses. For instance, NSCLC has been associated with high levels of miR-196a and miR-200b overexpression, with estimated fold changes of 23 and 37 times, respectively. (Guan et al. 2012; Vosa et al. 2013).

Methylation is an epigenetic gene regulation mechanism that can suppress the production of miRNAs in cancer cells (Wang et al. 2022).

Cao et al. discovered a unique miR-886-3p/PLK1 and TGF-b1 nexus DNA methylation in SCLC that controls tumor growth, invasion, and migration. As a result, it was proposed that, in some patients, SCLC is caused by hypermethylation of the miR-886-3p promoter gene, which results in a loss of miR-886-3p transcription. This loss then reverses the repression of PLK1 and TGF-b1 expression that miR-886-3p had previously imposed, activating the corresponding oncogenic pathways. These two active pathways work synergistically, in tandem with other essential alterations, to encourage cell proliferation and advance it to the more malignant stage of SCLC carcinogenesis. The decrease of miR-886-3p expression and hypermethylation of the miR-886 promoter and PLK1 are most likely potential biomarkers for adverse outcome prognosis and targets for SCLC therapy, due to the miR-886-3p-PLK1/TGF-b1 nexus regulation of SCLC aggressiveness. (Cao et al. 2013).

Several miRNA genes encoding members of the miR-34 family, miR-124a, miR-126, miR-9–3, miR-193a, miR-126, and miR-200c have been found to be transcriptionally controlled by methylation in NSCLC. In 65% of primary NSCLC cases, miR-9–3 methylation has been detected by Kitano et al. In turn, miR-34b and miR-126 have been found to be repressed by methylation in NSCLC, according to Watanabe et al. (Heller et al. 2018; Kitano et al. 2011; Watanabe et al. 2012).

According to research by Lujambio et al., methylation of the miR-9–3 gene in tumors is related to the development of lymph node metastases in NSCLC patients. In the study by Kim et al., it was discovered that methylation of the genes encoding miR-124–1 and miR-124–2 was associated with a shorter overall survival (OS) in NSCLC patients. Additionally, Tellez et al. examined the miR-196b gene methylation in sputum samples from stage I–III NSCLC patients and healthy controls and found that detection of this miRNAs was strongly correlated with the diagnosis of lung cancer (Lujambio et al. 2008; Kim et al. 2017a; Tellez et al. 2016).

miR-9–3 and miR-193a are specifically methylated in individuals with stage I-III NSCLC, according to the research by Heller et al. Additionally, they authors demonstrated that, in multivariate and univariate analyses, patients with lung squamous cell carcinomas (LSCC) with miR-9–3 methylation were characterized with significantly lower disease-free survival and shorter OS than miR-9–3-unmethylated LSCC patients. These findings imply that miR-9–3 methylation is a prognostic factor in LSCC. Therefore, it is possible that understanding the methylation status of miR-9–3 will enable personalization of care and treatment of LSCC patients following surgery (Heller et al. 2012).

Contrarily, only a small number of miRNA genes’ overexpression (e.g., miR-224 and let-7a-3) was linked to hypomethylation (Heller et al. 2018; Brueckner et al. 2007).

## The role of miRNAs in lung cancer development

It is well known that the aggressiveness of lung cancer, especially regarding its invasion and migration, often results in rapid disease progression despite standard treatment. miRNAs regulate multiple genes and different signal pathways. A growing body of research suggests that miRNAs in lung cancer act as tumor suppressors or oncomirs, modulating the expression of target mRNA to regulate cancer biology and promote tumor development (proliferating cancer), invasion, angiogenesis, and immune evasion. The mechanisms of cell development, cell proliferation, invasion, migration, apoptosis, and metastasis in lung cancer, both SCLC and NSCLC, have been shown in numerous studies to be significantly influenced by miRNAs (Zhong et al. 2021; Azizi et al. 2021). Selected miRNAs identified as regulators of lung cancer development are summarized in Table 1.

**Table 1:** miRNAs acting as tumor suppressor or oncogenes genes in lung cancer (SCLC and NCSLC) and their targets

miRNAs	Gene(s) and Protein(s)	Events
Tumor suppressor miRs (downregulation)		
SCLC		
miR-34	NA	Cell proliferation, invasion, metastasis in vitro
miR-138	H2AX	Cell growth, cell cycle, progression, DNA damage repair, cell proliferation
miR-126	SLC7A5	Cell proliferation
miR-450	IRF2	Cell proliferation, invasion, migration in vitro, tumor growth in vivo
miR-195	RAP2C	Tumor growth
NSCLC		
let-7	Ras, NIRF, HMGA2, STAT3, MYC, UHRF2, CASP3, CDK6, cyclinD2, CDC25A, KRAS, CCND1	Cell cycle, cell proliferation, apoptosis, poor prognosis (overall survival, early recurrence, tumor size)
miR-29b	MMP2, TGFB1	Invasion, MET
miR-98	TWIST1	EMT
miR-34 family	p53, MET, MYC, BCL-2 CCNE1, CDK4	Cell proliferation, apoptosis
miR-34a	SNAI-1	EMT
miR-124	CDH2	EMT
miR-128	VEGFA	Angiogenesis
miR-130a	HIF1A	Angiogenesis
miR-138	CD274	Immune escape
miR-140	CD274	Immune escape
miR-142	CD274	Immune escape
miR-148a	MMP15, ROCK1	Cell survival
miR-195	BIRC5, BCL-2, VEGFA	Apoptosis, angiogenesis
miR-199a	HIF1A	Angiogenesis
miR-200 family	ZEB1, ZEB2, CD274	EMT, MET, immune escape
miR-200b	FLT1, KDR	Angiogenesis, invasion, metastasis
miR-200c	HIF1A	Angiogenesis
miR-222	SOCS3	Cell survival
miR-451	PSMB8	Immune escape
miR-584	MMP14	Invasion
Oncomic (up-regulation)		
SCLC		
miR-25	Cyclin E2, CDK2	Cell proliferation, invasion
miR-141	KLF12	Neoangiogenesis
miR375	YAP-1, ITPKB	Cell growth
NSCLC		
miR-21	PTEN, Apaf1, Faslg, RhoB, COX 19, TPM1, PDCD4	Tumor progression, apoptosis
miR-130b	TIMP2	Invasion, angiogenesis
miR-182	CASP2	Apoptosis
miR-197	CSK1B	Immune escape
miR-212	PTCH1	Cell growth
miR-224	PPP2R2A, LATS2, SMAD4, TNFA1P1	Cell growth
miR-484	APAF1	apoptosis
miR-494	CASP2	apoptosis
miR-544a	CDH1	EMT

*NA* not assessed, *EMT* epithelial-to-mesenchymal transition, *MET* mesenchymal-to-epithelial transition

### Tumor suppressor

In SCLC tumors, several miRNAs directly target genes involved in the cell cycle, apoptosis, and DNA damage repair pathways, playing a role in control of cell proliferation and metastasis (Pandey et al. 2021).

It is widely recognized that neuroendocrine (NE) characteristics and an aggressive clinical course are characteristic for small cell lung cancer (SCLC). The impact of miRNAs in ASH1-induced NE characteristics of SCLC tumors has been examined in a number of studies. Nishikawa et al. found a significant positive association between the expression of miR-375 and ASH1. In lung cancer cells, ASH1 significantly upregulated miR-374, which was a signal strong enough to promote NE differentiation. Transcription of the miR-375 gene is activated when ASH1 binds three E-box elements (E1, E2, and E3) in the promoter region, as reported by Zhao et al. (Pandey et al. 2021; Zhao et al. 2012; Nishikawa et al. 2011).

On the other hand, miR-375 successfully reduced the expression of YAP-1. Along with YAP-1, miR-375 expression showed negative correlation with the expression of FZD8, ITGA10, ITPKB, LRP5, PIAS1, RUNX1, and YAP-1 (Nishikawa et al. 2011).

The expression of miR-34b/c was also downregulated in tumors compared to normal tissue. In SCLC tumors, miR-34 family members miR-34, miR-34b, and miR-34c are thought to act as a tumor suppressors. According to the results of Mizuno et al., miR-34b-3p was one of 35 miRNAs that were downregulated in primary SCLC tumors, as well as brain and liver metastases, compared to non-cancerous lesions from SCLC patients (Mizuno et al. 2017).

A number of reports suggest that miRNAs encoded by the let-7 family play a role in the inhibition of oncogenes including as rat sarcoma (RAS), myelocytomatosis oncogene (MYC), high mobility group Athook 2 (HMGA2), cyclin D2, cyclin-dependent kinase 6 (CDK6), and cell division cycle 25A (CDC25A) (Naidu and Garofalo 2015; Wu et al. 2019b; Zhao et al. 2018).

Let-7 was used as an example by Karube et al. to describe for the first time that a poor prognosis in lung cancer patients is associated with a lower level of dicer protein (Karube 2005). Moreover, let-7 miRNAs are underexpressed in the blood of NSCLC patients, according to the research by Jeong et al. Takamizawa et al., on the other hand, found that let-7 expression usually decreased in lung cancer both in vitro and in vivo. Additionally, regardless of the severity of the disease, down-regulated let-7 was correlated with poor post-operative survival (Jeong et al. 2011; Takamizawa et al. 2004).

Through direct targeting of K-Ras and cyclin D1, Kumar et al. have demonstrated that members of the let-7 family prevent the proliferation of NSCLC (Kumar et al. 2008).

Let-7a expression is also less common in lung adenocarcinomas, as reported by Zho et al. According to their findings, let-7a depletion may play a significant role in the emergence of lung cancer. Furthermore, reduction of cyclin D1 was found to be the mechanism through which let-7a therapy suppresses lung adenocarcinoma cell proliferation and triggers their apoptosis. Previous research on breast cancer has demonstrated that the interaction between filamin A and cyclin D1/cyclin-dependent kinase 4 can influence the tumor’s ability to migrate and invade (Zhao et al. 2018; Shen et al. 2020). The study of Xia et al. showed that patients with a low let-7 expression had a significantly shorter survival than those with a moderate expression. A similar correlation was observed in patients with high K-ras expression. The study confirmed that low expression of let-7 and high expression of Kras are correlated with the pathogenesis and prognosis of NSCLC. Low expression of let-7 is related to metastasis, vascular invasion and progression of NSCLC, and is an independent factor for poor prognosis (Xia et al. 2010).

The growth patterns of lung adenocarcinomas are also disturbed by the expression of miRNAs. According to the findings of Nadal et al. regarding histological growth patterns in lung adenocarcinoma, high expression levels of miR-27a, miR-212, and miR-132 were significantly associated with the presence of solid components in the tumor, whereas miR-30d was associated with lepidic patterns or mucinous invasive adenocarcinoma (Nadal et al. 2014).

In adenocarcinoma cells, Xiang et al. discovered that expression of miR-98, a member of the let-7 family, acts as a negative regulator of the HMGA2 (high mobility group A2) oncogene expression. This up-regulation of HMGA2 makes cells more sensitive to cisplatin (Xiang et al. 2013).

According to a number of studies, the miR-34 family was down-regulated in NSCLC cells compared to normal tissues. Furthermore, a worse prognosis correlated with a decrease in miR-34 expression. Bommer et al. reported that miR-34 expression was notably reduced in NSCLC. Moreover, the authors also reported that the miR-34 family may be the primary effector of the p53 tumor suppressor (Bommer 2007).

The members of the miR-200 family, including miR200a, miR200b, miR200c, miR-141, and miR-429, are similarly down-regulated in cancer cells. According to previous reports, the miR-200 family prevents invasion, metastasis, and the epithelial-to-mesenchymal transition (EMT). When miR-200c was overexpressed in NSCLC cancer cells, ZEB1 (zinc finger E-box-binding homeobox 1) expression was decreased, while E-cadherin expression was increased (Hurteau et al. 2007).

Furthermore, Jiang et al. have shown that, compared to the surrounding normal lung tissues, miR-125-3p/5p is down-regulated in NSCLC tumors. Patients with low miR-125a-3 expression and high miR-125a-5p expression had a higher likelihood of developing lymph node metastases (Jiang et al. 2010).

Recent research indicates that the expression of miR-124, initially attributed to neuronal cells, is notably downregulated in lung cancer. These reports also imply that DNA hypermethylation may be the root source of this downregulation. Furthermore, a number of studies have demonstrated that miR-124 has an oncogenic effect by directly inhibiting the expression of the transcription factor STAT3 (signal transducer and activator of transcription 3). STAT3, which may be a key target of miR-124, upregulates the expression of cyclin D1, survivin, Bcl-xL, and vascular endothelial growth factor (VEGF), which suppress cell apoptosis and promote tumor growth. Lee et al. reported that miR-124 suppresses cell growth and triggers apoptosis in NSCLC cells. According to their findings, miR-124 decreases lung cancer cells’ tumorigenic capacity through STAT3 downregulation. The study also confirmed a strong correlation between the TNM stage, differentiation grade, lymph node metastasis, and a decrease in miR-124 expression or an increase in STAT3 expression. MiR-124 was shown to be downregulated in patients with lymph node metastases or poor differentiation, which suggests that this downregulation might occur as a result of tumor growth or, potentially, the development of metastatic potential (Li et al. 2015).

### Oncogenic miRNA

miR-21 was the first to be recognized as an oncogenic miRNA, among the numerous miRNAs that have since been linked to the development of cancer (Rhim et al. 2022).

miR-21 expression was elevated in NSCLC with R175H- and R248Q-mutant p53, with Zhou et al. indicating it as a poor prognostic factor (Zhou et al. 2020).

miR-155 was identified as another poor prognostic factor. Overall survival rate was poorer in cancer patients with higher expression of miR-155a. Through the downregulation of SOCS1, SOCS56, and PTEN, miR-21 and miR-155 may both promote NSCLC cell proliferation and invasion (Xu et al. 2018; Singh et al. 2015).

Between 15 and 30% of patients with lung cancer experience bone metastases. MicroRNAs are implicated in lung cancer bone metastases, according to a number of studies. A report by Guo et al. established the role of miR-21 in H2170 NSCLC tumors, acting in suppression of apoptosis and boosting cell proliferation. Additionally, miR-21 promotes tumor growth through targeting PDCD4, which controls osteoclastogenesis. Furthermore, STAT3 also targets miR-21, with both STAT knockdown and miR-21 downregulation resulting in reduction of brain metastasis-initiating cell self-renewal and migration (Guo et al. 2015).

In another study, overall survival (OS) was linked to low levels of miR-548j-5p and high levels of miR-93-3p. Furthermore, miR-494-3p up-regulation has been associated with worse lung cancer patient outcomes and lung cancer progression (Faversani et al. 2017).

## The role of miRNAs in lung cancer diagnosis

Numerous studies have demonstrated that identifying different risk factors and biomarkers may aid in a better understanding of the cellular and molecular changes involved in the progression of cancer. Additionally, it would be more beneficial to monitor lung cancer patient therapy if a potent diagnostic and prognostic biomarkers were used. Hence, miRNAs have the potential to serve as novel predictive, therapeutic, and diagnostic biomarkers (Zhou et al. 2017; Thakur and Gadgeel 2016). High levels of specificity, sensitivity, and predictive power are the desirable characteristics of biomarkers. There are several inherent qualities of miRNAs that make them perfectly suited for that role (Zhou et al. 2017; Langevin et al. 2015).

The first benefit is that they are more specialized than typical gene expression analyses, and it has been demonstrated that miRNAs expression profiles differ between cancer types depending on the diagnosis and the tumor’s developmental stage (Larrea et al. 2016; Lu et al. 2005).

Secondly, miRNAs are extremely stable in bodily fluids because they are not affected by endogenous RNases. Additionally, formalin-fixed paraffin-embedded (FFPE) material may be regularly used to obtain them (Larrea et al. 2016; Lawrie et al. 2007), while boiling, pH fluctuations, frequent freeze–thaw cycles, and enzyme- or chemical-induced fragmentation do not seem to harm miRNAs (Larrea et al. 2016; Mitchell et al. 2008; Cortez et al. 2011; Mo et al. 2012). Thirdly, the problem of tumor heterogeneity can be solved by analysis of circulating miRNAs, which gather all pathologic signals from various tumor parts or metastatic sites (Mo et al. 2012).

Moreover, it is crucial that miRNAs are widely accessible, as they can be found in pleural fluid, blood, colostrum, urine, and other bodily fluids. The examination of miRNAs can be repeated frequently throughout therapy because it is reasonably affordable and non-invasive. Because of all these factors, miRNA analysis is an effective tool for tracking the progression of cancer (Mo et al. 2012).

Numerous miRNAs are currently being employed as a lung cancer diagnosis tool. A serum-based miRNAs test called miR-test, for instance, examines the signatures of 13 different miRNAs, including miR-92a-3p, miR-30b-5p, miR-191-5p, miR-484, miR-328-3p, miR-30c-5p, miR-374a-5p, let-7d-5p, miR-331-3p, miR-29a-3p, miR148a-3p, miR-223-3p, and miR-140-5p. The miR-test has a diagnostic accuracy, sensitivity, and specificity of 74.9%, 77.8%, and 74.8%, respectively, in patients with high-risk lung cancer. Furthermore, using the expression ratio of 24 miRNAs specific for lung cancer, the miRNAs’ signature classifier (MSC) commercial kit has reported sensitivity, specificity, positive predictive value, negative predictive value, and positive probability ratios of 87%, 81%, 27%, 98%, and 4.67%, respectively (Han and Li 2018; Montani et al. 2015; Sozzi et al. 2014).

Lung cancer subtypes could also be distinguished using miRNAs. Lung cancer can be histologically categorized as non-small-cell lung cancer (NSCLC) and small-cell lung cancer (SCLC). The latter, which can be further split into adenocarcinoma, squamous cell carcinoma, and large-cell carcinoma, accounts for more than 80% of instances of lung cancer (Bishop et al. 2010; Gyoba et al. 2016).

Numerous research results back up the idea that miRNAs could serve as a marker for non-small cell lung cancer (NSCLC). The let-7 miRNA is one among the more well-known examples. Human let-7 gene expression levels have been observed to vary among different adult tissues, with the lung being one of the tissues with the most abundant let-7 expression (Takamizawa et al. 2004; Pasquinelli et al. 2000).

According to research by Takamizawa et al., let-7 overexpression inhibits lung cancer cell proliferation, and let-7 expression is strongly inversely correlated with postoperative survival time. These data collectively imply that decreased expression of let-7 may contribute to the development of lung malignancies (Takamizawa et al. 2004).

miR-205 is known to be specific for squamous cell carcinomas, and miR-124a for adenocarcinomas (Han and Li 2018; Lebanony et al. 2009).

In other research, Zhang et al. found that squamous cell carcinoma is characterized by high expression of miR-205, miR-93, miR-221, and miR-30e, while adenocarcinoma exhibits high expression of miR-29b, miR-29c, let-7, miR-100, and miR-125a-5p. Moreover, according to reports, miR-375 and miR-21-5p demonstrate elevated expression levels in SCLC (Nishikawa et al. 2011; Zhang et al. 2012; Demes et al. 2016).

Making the distinction between primary and metastatic lung cancers is another crucial stage in the cancer diagnosis process. According to Barshack et al., high expression of miR-126 was found in metastatic cancers, whereas high expression of miR-182 was characteristic for initial lung malignancies. Moreover, elevated expression miR-552 and miR-592 have been utilized to distinguish primary lung adenocarcinoma from metastatic colorectal cancer (Barshack et al. 2010; Kim et al. 2014).

## Lung cancer therapy—the role of miRNAs

The response to chemotherapy, radiation, and targeted therapy can be influenced by miRNAs (Lu et al. 2018b). Hence, miRNAs appear to be suitable to develop useful therapeutic targets, reversing cancer characteristics or making tumors more responsive to treatment. A single miRNA has the ability to affect the activity of a biological signaling pathway or coordinately regulate numerous targets in a separate pathway, as each miRNA simultaneously targets several genes (Backes et al. 2016; Garzon et al. 2010; Kasinsk and Slack 2011).

### Chemotherapy

The most often used lung cancer treatments, whether it is SCLC or NSCLC, are platinum-based. miRNAs can also influence the susceptibility or resistance to chemotherapy. According to Yu et al., increased miR-106b expression decreased polycystin 2 (PKD2) levels and consequently decreased P-glycoprotein, which mediates the efflux of several anticancer drugs. As a result, cisplatin sensitivity was raised in an NSCLC cell line with the up-regulation of miR-106b (Yu et al. 2017).

Wu et al. demonstrated that mir-503 suppressed many resistance-associated proteins, including as MDR1, MRP1, ERCC1, survivin, and Bcl-2, to impede the drug efflux mechanism and improve cisplatin sensitivity (Wu et al. 2014).

On the other hand, enhanced MDR1, MRP1, ERCC1, survivin, and Bcl-2 were linked to upregulated miR-196, leading to drug efflux and cisplatin resistance. By focusing on PEBP4- and RKIP-mediated EMT, both miR-15b and miR-27a contributed to cisplatin treatment resistance (Li et al. 2016; Zhao et al. 2015).

### Radiation Therapy

Numerous investigations have found a connection between radiation and clinical uses of miRNAs. miRNAs are known to control the tempo of cell division and apoptosis, interfere with the organism’s ability to repair damaged DNA, and promote tumor angiogenesis.

Chen et al., in a bioinformatical analysis, revealed that miR-98-p5, miR-302e, miR-495-3p, and miR-613 might be used as a predictor of radiation response in NSCLC (Chen et al. 2016).

In turn, Cortez et al. demonstrated that, through suppression of oxidative response genes and prevention of DNA repair, miR-200c could improve the effectiveness of radiation on lung cancer. Similar effects were demonstrated by miR-449a, miR-138, miR-25, and let-7 (Cortez et al. 2014).

Furthermore, miR-495-3p showed a larger abundance in the plasma of participants with a complete response to the treatment than those with a less favorable reaction, according to Chen et al. (Chen et al. 2016).

Finally, increased levels of miR-494-3p, miR-493-5p, and miR-495-3p were found in patients with lung cancer whose overall survival (OS) was greater than 6 months in a different investigation (Chen et al. 2016).

### Immunotherapy

The possibility to blocking immunological checkpoints has completely changed how cancer is treated, as immunotherapy has emerged as a viable treatment option. Immunological checkpoints are inhibitory pathways that typically regulate immune tolerance and mitigate tissue damage following inflammatory response (Pardol 2012).

Immune checkpoint inhibition is a new treatment option for non-small cell lung cancer (NSCLC) that usually offers patients much better prognoses. In patients with NSCLC, checkpoint inhibitor response rates were estimated at around 20% (Pardol 2012).

According to a number of clinical studies, immunological checkpoint inhibitors cause a response in 20% to 30% of unselected NSCLC patients, with some of these patients achieving persistent disease control with minor side effects (Brahmer et al. 2015; Borghaei et al. 2015).

Additionally, it has been demonstrated that tumor-derived miRNAs can alter the behavior of cells in the tumor microenvironment (Suzuki et al. 2015) and may be crucial for the interaction of immune and tumor cells. Specific miRNAs are connected to immune cells that are active in the tumor stroma and likely affect the tumor microenvironment modifications (Kuninty et al. 2016).

In NSCLC patients receiving nivolumab therapy, Halvorsen et al. discovered a profile of seven circulating miRNAs, including mir-215-5p, miR-411-3p, miR-493-5p, miR-494-3p, miR-495-3p, miR-548j-5p, and miR-93-3p, that are strongly related with improved overall survival (Halvorsen et al. 2018).

### Molecular target therapy

#### EGRF-tyrosine kinase inhibitor

Recent research revealed that epigenetic mechanisms may play a significant role in treatment resistance, at least in some circumstances. Numerous studies have found a link between altered miRNA expression and susceptibility to erlotinib and gefitinib (Garofalo et al. 2011; Shen et al. 2014). Furthermore, some research demonstrated that miR-21 causes gefitinib resistance in NSCLC by inhibiting PTEN and activating ALK and ERK (Li et al. 2014).

Other studies have shown that miR-133b is elevated when erlotinib is administered as the second- or third-line therapy for NSCLC, with this correlation associated with a prolonged progression-free survival (Bisagni et al. 2018). Although it has been established that the MET oncogene has a role in both de novo and acquired resistance of non-small cell lung malignancies to tyrosine kinase inhibitors (TKIs), the specific mechanism by which MET overexpression results in TKI-resistant NSCLC is still unknown (Bisagni et al. 2018).

Tyrosine kinase receptor effects on miRNAs were investigated by Garofalo et al. In contrast to miR-103 and miR-203, which were only regulated by MET, they found that miR-30b, miR-30c, miR-221, and miR-222 were affected by both the epidermal growth factor (EGF) and MET receptors. By inhibiting the expression of the genes encoding BCL2-like 11 (BIM), apoptotic peptidase activating factor 1 (APAF-1), protein kinase C (PKC-), and sarcoma viral oncogene homolog, they demonstrated that these miRNAs played significant roles in gefitinib-induced apoptosis and epithelial-mesenchymal transition of NSCLC cells in vitro and in vivo (SCR, non-receptor cytoplasmic tyrosine kinase). The above results imply that targeting particular miRNAs could offer a promising therapeutic strategy for the management of NSCLC (Garofalo et al. 2011; Shen et al. 2014; Li et al. 2014; Bisagni et al. 2018; Wang et al. 2012; Zhong et al. 2010; Kitamura et al. 2014; Cao et al. 2012; Cufi et al. 2013; Bryant et al. 2012; Shaw et al. 2009).

#### ALK-TKI

One of the newest molecular targets in NSCLC is the oncogene that results from the EML4-ALK fusion. The fusion, which was first described in 2007 (Soda et al. 2007; Rikova et al. 2007), is caused by a small inversion on chromosome 2p and results in the expression of a chimeric tyrosine kinase in which the intracellular kinase domain of anaplastic lymphoma kinase (ALK) is fused to the N-terminal half of echinoderm microtubule-associated protein-like 4 (EML4) (Chiarle et al. 2008).

Both in vitro and in vivo, EML4-ALK exhibits strong carcinogenic potential (Soda et al. 2007, 2008). Small-molecule inhibitors of ALK can effectively stop this activity (Soda et al. 2008; Koivunen et al. 2008), supporting the idea that EML4-ALK functions as the primary initiator of lung carcinogenesis.

The miRNA expression profile of lung tumors with ALK rearrangements was distinct from lung cancers with EGFR and KRAS mutations. In addition, miR-342-3p and let-7e expression levels were downregulated, and E-cadherin was lost in lung cancers with ALK rearrangements. These traits might shed light on the biological mechanisms underlying the relatively aggressive nature of lung cancers with ALK rearrangements and suggest potential therapeutic approaches to overcome ALK inhibitor resistance and stop EMT-mediated metastasis (Kim et al. 2017b; Kwak et al. 2010).

In between 3 and 7% of patients with NSCLC, there are some ALK rearrangements. Young age, no smoking history or a history of light smoking, and adenocarcinoma histology are the main characteristics of ALK-positive lung cancer patients (Soda et al. 2008; Shaw et al. 2013). Crizotinib, an ALK inhibitor, is very effective in treating patients with advanced ALK-positive NSCLC, with an objective response rate of about 60% and a median progression-free survival of 8 to 10 months (Kwak et al. 2010; Shaw et al. 2013; Li et al. 2017).

Circulating miRNAs were investigated by Li et al. as diagnostic and prognostic indicators for Crizotinib-treated ALK-positive lung cancer. The authors discovered a novel panel of three miRNAs (miR-28-5p, miR-362-5p, and miR-660-5p) that had good specificity and sensitivity for identifying ALK-positive from ALK-negative NSCLC. Their findings imply that this panel of three miRNAs has a lot of potential as an auxiliary diagnostic and that dynamic miRNAs biomarker monitoring may be beneficial for ALK-positive NSCLC patients treated using Crizotinib (Li et al. 2017).

Moreover, Yun et al. argued that techniques targeting epigenetic pathways constituted a potentially viable method for overcoming acquired resistance to cancer therapy (Yun et al. 2018). They also noted that enhancer remodeling and altered production of miRNAs played essential roles in cancer drug resistance.

Histone H3 lysine 27 acetylation results in the development of resistance to ALK inhibitors in ALK-positive lung tumors (H3K27ac). When resistance was acquired, H3K27ac underwent significant modification, and miRNA and mRNA expression changed as a result of enhancer remodeling. Target genes like AXL were activated in a manner that was associated with decreased H3K27ac levels and decreased miR-34a expression. On resistant cells, xenografts, and EML4-ALK transgenic mice, panobinostat, a pan-histone deacetylase inhibitor, altered the H3K27ac profile and activated tumor-suppressor miRNAs like miR-449, another member of the miR-34 family, which together had an antiproliferative effect. The inhibition of miR-34a or miR-449a and the activation of AXL were shown to be mutually exclusive of secondary mutations in ALK in a matched examination of patient samples before and after treatment with ALK inhibitors. According to these findings, enhancer remodeling and altered miRNA expression are crucial factors in cancer treatment resistance, and techniques that target epigenetic pathways may be an efficient way to combat acquired resistance to cancer therapy (Yun et al. 2018).

## Completed and ongoing clinical trials

miRNAs regulate gene expression post-transcriptionally and are often downregulated in cancer. Restoring miRNA levels has long been known to suppress tumor growth, with numerous studies demonstrating such results in preclinical models. Hence, due to these promising results, there are already some clinical trials based on miRNA replacement therapy.

One of the first preclinical models used to investigate a miRNA mimic-based therapy was lung cancer. Since then, let-7, miR-34, and miR-15/16 families have been the subject of a number of studies (Fortunato et al. 2014; Sayed et al. 2021). As research findings were not yet sufficiently validated, miRNAs are not frequently used in the clinical treatment of lung cancer. The ClinicalTrials.gov database (Home—ClinicalTrials.Gov. 2022) is the most recent source of information on finished and continuing clinical trials employing liquid biopsy, including miRNAs in lung cancer.

Table 2 lists studies on miRNAs in lung cancer that have been completed, whereas Table 3 lists studies that are still in progress.

**Table 2:** Completed clinical studies on miRNAs (based on ClinicalTrials.gov (accessed on 30 November 2022))

No	NCT no	Study title	Cancer	Location	Participants (Active/original)
	NCT03452514	Addition of microRNA Blood Test to Lung Cancer Screening Low Dose CT	Lung cancer	Massachusetts General Hospital, Boston, Massachusetts, USA Brigham and Women’s Hospital, Boston, Massachusetts, USA Beth Israel Deaconess Medical Center, Boston, Massachusetts, USA Lahey Hospital & Medical Center, Burlington, Massachusetts, USA	479/479
	NCT02445924	MicroRNA Genetic Signature in NSCLC Egyptian Patients	Non-small cell lung cancer	Chest Department, Faculty of Medicine, Tanta University, Tanta, Gharbia, Egypt	40/40
	NCT03293433	Quantification of microRNAs in Diagnosis of Pulmonary Nodule	Pulmonary cancer	University Hospital of la Réunion, France University Hospital of Toulouse, Toulouse, France	103/103
4	NCT03741829	TS Overexpression in SCLC: Mechanism and Therapeutic Targeting	Small cell lung cancer	Wake Forest University Health Sciences Wilson-Salem, North Carolina, USA	12/12
5	NCT00897234	Blood Samples From Patients From Patients With Non-Small Cell Lung Cancer and From Healthy Volunteers	Lung cancer	Masonic Cancer Center at University of Minnesota, Minneapolis, Minnesota USA	84/84
6	NCT00864266	Biological Factors Predicting Response to Chemotherapy in Advanced Non Small Cell Lung Cancer	Non-small cell lung cancer	Department of Pneumology Clinique Saint-Luc, Bouge, Belgium Department of Intensive Care Unit and Thoracic Oncology Institut Jules Bordet, Brussels, Belgium Service de Pneumologie Hôpital Erasme, Brussels, Belgium Department of Pneumology Hôpital Saint-Joseph, Gilly, Belgium Hôpital Ambroise Paré, Mons, Belgium Department of Pneumology Centre Hospitalier de Mouscron, Mouscron, Belgium	70/70
7	NCT02837809	Early Lung Cancer Detection in High Risk Individuals	Lung cancer	Fondazione IRCCS Istituto Nazionale dei Tumori, Milan, Italy	4099/4099
8	NCT00840125	Study of Erlotinib With Docetaxel in Selected Non Small Cell Lung Cancer Patients in First Line Treatment	Non-small cell lung cancer	Meir Medical Center, Kfar Saba, Israel	4/40
9	NCT02369198	MesomiR 1: A Phase I Study of TargomiRs as 2nd or 3rd Line Treatment for Patients With Recurrent MPM and NSCLC	Malignant pleural mesothelioma Non-small cell lung cancer	Concord Repatriation General Hospital, Sydney, New South Wales, Australia Chris O'Brien Lifehouse, Sydney, New South Wales, Australia Northern Cancer Institute, Sydney, New South Wales, Australia	27/30
10	NCT00926640	A Phase I Study of Belinostat in Combination With Cisplatin and Etoposide in Adults With Small Cell Lung Carcinoma and Other Advanced Cancers	Carcinoma neuroendocrine Small cell lung cancer carcinoma Malignant epithelial neoplasms	National Institutes of Health Clinical Center, 9000 Rockville Pike, Bethesda, Maryland, USA	28/44
11	NCT01779388	Bronchoscopy Assisted by Electromagnetic Navigation (EMN) in the Diagnosis of Small Pulmonary Nodules	Pulmonary nodule Cm	Institut Jules Bordet, Brussels, Belgium	120/120
12	NCT02387307	A Study of rSIFN-co in Subjects With Advanced Solid Tumors	Carcinoma, non-small-cell lung Carcinoma, Renal cell Melanoma Carcinoma, hepatocellular Colon cancer	National Cancer Centre Singapore, Singapore	42/42
13	NCT02169271	Acetylsalicylic Acid Compared to Placebo in Treating High-Risk Patients With Subsolid Lung	Multiple pulmonary nodules	M D Anderson Cancer Center, Houston, Texas, USA European Institute of Oncology, Milano, Italy	109/128
14	NCT02194231	ATREUS—Phase II Study on the Activity of Trabectedin in Patients With Malignant Pleural Mesothelioma (MPM)	Malignant pleural mesothelioma	Azienda ospedaliera ss. Antonio e Biagio e Cesare Arrigo, Alessandria, AL, Italy Cliniche Humanitas Gavazzeni, Bergamo, BG, Italy Azienda Ospedaliera Universitaria Policlinico Sant'Orsola Malpighi, Bologna, Bo, Italy P.O. Spedalli Civili, Brescia, BS, Italy Azienda Ospedaliera S. Gerardo di Monza, Monza, MB, Italy Istituto Clinico Humanitas, Rozzano, MI, Italy •Istituto Oncologico Veneto—IOV, Padova, PD, Italy •Azienda Ospedaliro-Universitaria di Parma, Parma, Italy	145/145

**Table 3:** Ongoing clinical studies on miRNAs (based on ClinicalTrials.gov (accessed on 30 November 2022))

No	NCT no	Study title	Cancer	Location	Participants (Estimated)
1	NCT02247453	Plasma microRNA Profiling as First Line Screening Test for Lung Cancer Detection: a Prospective Study	Lung cancer	Fondazione IRCCS Istituto Nazionale dei Tumori, Milan, Italy	4119
2	NCT03074175	Plasma miRNAs Predict Radiosensitivity of Different Fractionation Regimes in Palliative Radiotherapy for Advanced Non-small Cell Lung Cancer Multicenter Controlled Study. (RadimiR-01)	Advanced NSCLC	Xinqiao Hospital of Chongqing, Chongqing, China	240
3	NCT04427475	Prediction of Immunotherapeutic Effect of Advanced Non-small Cell Lung Cancer	NSCLC	Cancer Hospital Fudan University Shanhai, Shanghai, China	200
4	NCT01240369	Association Between VEGF-C and miRNA Clinical Non-small Cell Lung Cancer and Esophagus Squamous Cell Carcinoma	NSCLC, ESCC		250
5	NCT04323579	Validation of Multiparametric Models and Circulating and Imaging Biomarkers to Improve Lung Cancer EARLY Detection	Lung cancer	Istituto Clinico Humanitas, Rozzano, Milano, Italy	2000
6	NCT04629079	Improving the Early Detection of Lung Cancer by Combining Exosomal Analysis of Hypoxia With Standard of Care Imaging	Lung cancer	Borthwick Research Unit, Lister Hospital, Stevenage, UK	800
7	NCT03397355	Monitoring the Changes of Tumor-related Biomarkers Before and After Pulmonary Nodule Biopsy	Lung cancer	China-Japan Friendship Hospital, Beijing, Beijing, China	1000
8	NCT04965129	Biopsy Supplementation of n-3 PUFA in the Modulation of Lean Mass in Patients With Lung Cancer Receiving a High-protein Diet	Lung cancer	Federal University of Rio de Janeiro, Rio de Janeiro, RJ, Brazil	50
9	NCT02301858	Identification of Predictive and Prognostic Markers for Lung Cancer With Metastases	Metastatic lung cancer	Vestfold Hospital Trust, Tønsberg, Norway	100
10	NCT04315766	Optimised Lung Cancer Screening to Prevent Cardiovascular and Pulmonary Diseases Coupled With Primary Prevention	Lung cancer	Istituto Clinico Humanitas, Rozzano, Milano, Italy	2000
11	NCT03233724	Oral Decitabine and Tetrahydrouridine as Epigenetic Priming for, Pembrolizumab-Mediated Immune Checkpoint Blockade in Patients With Inoperable, or Unresectable Locally Advanced or Metastatic Non-Small Cell Lung Cancers and Esophageal Carcinomas	Advanced or metastatic non-small cell lung cancers and esophageal carcinomas	National Institutes of Health Clinical Center, Bethesda, Maryland, USA	85
12	NCT03721120	Evaluation of the Feasibility and Clinical Relevance of Liquid Biopsy in Patients With Suspicious Metastatic Lung Cancer	Metastatic lung cancer	Centre Hospitalier de Bayeux, Bayeux, France Hopital Louis Pradel, Bron, France Centre François Baclesse, Caen, France Centre Maurice Tubiana, Caen, France Infirmerie Protestante, Caluire et Cuire, France Centre Hospitalier Public du Cotentin, Cherbourg, France CH Les Oudairies, La Roche-sur-Yon, France Hôpital Privé Jean Mermoz, Lyon, France Centre Leon Berard, Lyon, France Groupe Hospitalier de la région de Mulhouse et Sud-Alsace, Mulhouse, France Centre Hospitalier de Bayeux, Bayeux, France Hopital Louis Pradel, Bron, France Centre François Baclesse, Caen, France Centre Maurice Tubiana, Caen, France Infirmerie Protestante, Caluire et Cuire, France Centre Hospitalier Public du Cotentin, Cherbourg, France CH Les Oudairies, La Roche-sur-Yon, France •Hôpital Privé Jean Mermoz, Lyon, France •Centre Leon Berard, Lyon, France Groupe Hospitalier de la région de Mulhouse et Sud-Alsace, Mulhouse, France and 6 more	286
13	NCT02511288	Liquid Biopsies in Patients Presenting Non-small Cell Lung Cancer	Non-small cell lung	Centre Hospitalier Annecy Genevois, Annecy, France CH Fleyriat, Bourg-en-Bresse, France Hôpital Louis Pradel, Bron, France CHU Grenoble Alpes, Grenoble, France Centre Léon Bérard, Lyon, France CHRU de Saint-Etienne, Saint-Priest-en-Jarez, France Institut de Cancérologie Lucien Neuwirth, Saint-Priest-en-Jarez, France Hôpital Nord Ouest, Villefranche-sur-Saône, France	900
14	NCT03824327	Papaverine and Stereotactic Body Radiotherapy (SBRT) for Non Small Cell Lung Cancer (NSCLC) or Lung Metastases	Lung non-small cell carcinoma	Ohio State University Comprehensive Cancer Center, Columbus, Ohio, United States	24
15	NCT04515004	Leucoselect Phytosome for Neoadjuvant Treatment of Early Stage Lung Cancer	Early-stage lung cancer (I and II)	New Mexico VA Health Care System, Albuquerque, NM, Albuquerque, New Mexico, United States	30
16	NCT01560195	A Study of Pegylated rhG-CSF as Support to Advanced Non-Small-Cell Lung Cancer (NSCLC) Patients Receiving Chemotherapy	NSCLC	Shanghai Pulmonary Hospital, Shanghai, Shanghai, China	150
17	NCT05136846	Papaverine in Combination With Chemoradiation for the Treatment of Stage II-III Non-small Cell Lung Cancer	Locally advanced lung non-small cell carcinoma	Ohio State University Comprehensive Cancer Center, Columbus, Ohio, USA	28
18	NCT04392505	Durvalumab(MEDI4736) After chemoRadioTherapy(DART) for NSCLC-a Translational and Biomarker Study	Non-small cell carcinoma stage III	North Estonia Medical Centre, Tallinn, Estonia Oulu University Hospital, Oulu, Finland Tampere University Hospital, Tampere, Finland Turku University Hospital, Turku, Finland National Cancer Institute, Vilnius, Lithuania Haukeland universitetssykehus, Bergen, Norway Oslo University Hospital, Oslo, Norway Stavanger University Hospital, Stavanger, Norway Universitetssykehuset i Nord-Norge, Tromsø, Norway St. Olavs Hospital, Trondheim, Norway	100
19	NCT01629498	Image-Guided, Intensity-Modulated Photon or Proton Beam Radiation Therapy in Treating Patients With Stage II-IIIB Non-small Cell Lung Cancer	Non-small cell carcinoma stages II–III	M D Anderson Cancer Center, Houston, Texas, USA	100
20	NCT05311709	Sotorasib in Advanced KRASG12C-mutated Non-small Cell Lung Cancer Patients With Comorbidities	Non-small cell carcinoma stages III–IV	Vestre Viken Health Trust, Drammen, Viken, Norway	100
21	NCT03108677	Circulating Exosome RNA in Lung Metastases of Primary High-Grade Osteosarcoma	Lung metastases Osteosarcoma	Ruijin Hospital Shanghai Jiao Tong University School of medicine, Shanghai, Shanghai, China	40
22	NCT05000710	Concomitant Radiotherapy, Tremelimumab & Durvalumab for Advanced NSCLC Patients Progressing on First-line Immunotherapy	Metastatic or locally advanced NSCLC	Sheba Medical Centre, Ramat Gan, Israel	29

Clinical trials have been completed and are currently being conducted with the goal of improving standards for miRNA detection and characterization technologies.

There are sixteen current clinical trials listed in the ClinicalTrials.gov database: seven phase 1 trials (3 completed, 4 recruiting), ten phase 2 trials (3 finished, 5 recruiting, and 2 not recruiting), and just one clinical phase 3 study with no known status (Home—ClinicalTrials.Gov. 2022).

Early data from two phase I clinical trials have shed light on the effectiveness of innovative miRNA-based therapeutic strategies. The trial comprised patients for whom prior therapies had failed. Patients who had received prior therapies in full or part were included in the phase I of the study.

MesomiR 1: A TargomiRs Phase I Study Patients with NSCLC and malignant pleural mesothelioma were included in MesomiR 1 (NCT02369198, A Phase I Study of TargomiRs), a first-in-man, phase I clinical trial, to evaluate the safety and activity of TargomiRs as the second and third line of treatment. TargomiRs are targeted minicells carrying a microRNA mimic. They are made up of three parts: (a) A microRNA mimic based on miR-16, as several different forms of cancer have been linked to the miR-16 family’s role as a tumor suppressor. The mimic is a synthetic double-stranded RNA molecule of 23 base pairs; (b) EDV—a drug delivery system, EDVs are non-living bacterial minicells (nanoparticles). They serve as impermeable micro-reservoir carriers that enable effective packing of a variety of various medicines, proteins, or nucleic acids; (c) targeting moiety, an anti-EGFR bispecific antibody directs the EDVs to cancer cells that express EGFR (Reid et al. 2016).

MesomiR-1 began in December 2014 and finished in January 2017. TargomiRs has been administered to 27 people at various doses and times. Overall, TargomiR medication has been accepted well by patients and has been proved to be secure. Future studies of MesomiR-1 hold considerable potential given the disease control rate of 65% observed. These positive findings are supportive of a phase 2 clinical trial that aims to assess the effectiveness of mesomiR-1 therapy either alone or in conjunction with conventional chemotherapy (Reid et al. 2016; Zandwijk et al. 2017).

The first miRNAs replacement medication to be utilized in a therapeutic setting was the miR-34a mimic, MRX34. A 23-nt synthetic, double-stranded miR-34a mimic called MRX34 is contained inside a liposomal nanoparticle. The MRX34, microRNA miR-RX34 liposomal injection, was the subject of a multicenter phase I research (NCT01829971) to assess its safety, pharmacokinetics, and pharmacodynamics in patients with SCLC, NSCLC, primary liver cancer, as well as other selected solid tumors and hematologic malignancies. However, five severe immunological reactions that resulted in the deaths of four participants caused it to be halted and ultimately canceled. The investigation could not conclusively determine whether the clinical effects (both toxicity and anticancer activity) of MRX34 are linked to the non-specific inflammatory effects of MRX34, the gene-suppressing activity of the miR-34a nucleotide, or some other mechanism (Agostini and Knight 2014; Hong et al. 2020).

There are just a few miRNAs-based applications that have been integrated into clinical practice, despite extensive and prolific research efforts in preclinical and clinical contexts. Serious adverse effects in the MRX34 study, including as sepsis, hypoxia, cytokine release syndrome, and liver failure, tended to happen after the daily MRX34 infusion had stopped. The results suggest that the toxicity could have been immune system related. Additionally, no negative effects were seen in MRX34 preclinical investigations using animal models. To prevent inducing an unintended immune response, novel miRNAs delivery techniques will need to be created in the future (Agostini and Knight 2014; Hong et al. 2020).

## Conclusion

The use of miRNAs as biomarker in lung cancer is an intriguing approach in the liquid biopsy era. miRNAs are demonstrated to be involved in the pathogenesis, diagnosis, and prognosis in lung cancer. However, the lack of reproducibility between different studies is the main barrier in using miRNAs for diagnostic purposes. The inter-individual variability in the level of miRNAs in the serum may be one cause of such discrepancies. The variability of technical aspects between different tests, e.g., RNA purification procedures also needs to be addressed. Furthermore, discrepancies can also be attributed to statistical methodologies, etc.

More comprehensive studies are also needed to demonstrate whether lung cancer patients can benefit from miRNAs targeted therapy. Nevertheless, available data of their features strongly suggest that miRNAs can act as an innovative and precise tool in clinical oncology.

## Data Availability

Not applicable.
